# Energy Absorption in Actual Tractor Rollovers with Different Tire Configurations

**DOI:** 10.3390/ijerph18126517

**Published:** 2021-06-17

**Authors:** Enrico Capacci, Bruno Franceschetti, Andrew Guzzomi, Valda Rondelli

**Affiliations:** 1 Department of Agricultural and Food Sciences, University of Bologna, Viale G. Fanin 50, 40127 Bologna, Italy; enrico.capacci@unibo.it (E.C.); bruno.franceschetti@unibo.it (B.F.); 2 School of Engineering, The University of Western Australia, 35 Stirling Highway, Crawley, WA 6009, Australia; andrew.guzzomi@uwa.edu.au; 3 The Institute of Agriculture, The University of Western Australia, 35 Stirling Highway, Crawley, WA 6009, Australia

**Keywords:** operator safety, ROPS, OECD, available energy, agriculture, silent-block

## Abstract

In order to better understand the complexities of modern tractor rollover, this paper investigates the energy absorbed by a Roll-Over Protective Structure (ROPS) cab during controlled lateral rollover testing carried out on a modern narrow-track tractor with a silent-block suspended ROPS cab. To investigate how different tractor set-ups may influence ROPS and energy partitioning, tests were conducted with two different wheel configurations, wide (equivalent to normal ‘open field’ operation) and narrow (equivalent to ‘orchard/vineyard’ operation), and refer to both the width of the tires and the corresponding track. Dynamic load cells and displacement transducers located at the ROPS-ground impact points provided a direct measurement of the energy absorbed by the ROPS cab frame. A trilateration method was developed and mounted onboard to measure load cell trajectory with respect to the cab floor in real-time. The associated video record of each rollover event provided further information and opportunity to explain the acquired data. The narrow tire configuration consistently subjected the ROPS cab frame to more energy than the wide tire arrangement. To better evaluate the influence of the ROPS cab silent-blocks in lateral rollover, static and dynamic tests were performed. The results confirm that tires influence the energy partition significantly and that further understanding of silent-blocks’ dynamic performance is warranted.

## 1. Introduction

For decades, Roll-Over Protective Structures (ROPS) have been mandatory on tractors in most countries. Despite this, serious injuries and fatalities of ROPS fitted tractor users still occur and hence, tractor rollover safety is an ongoing matter of concern. Conservative estimates [1] place the number of lives lost annually to tractor rollover in the U.S. at 120. The continued occurrence of fatalities [2,3,4] has led researchers and standards committees to question the relevance of the official procedures for testing modern tractor ROPS strength. Rollover research investigating ROPS energy absorption requirements began in the 1950s. Moberg [5], Chisholm [6,7,8,9] and Schwanghart [10,11] were pioneering researchers who advanced the field. Their publications explain various methodologies used in experimental work from which theoretical models were conceived and international testing procedures derived [12]. As a result of the efforts of those pioneering researchers, the normalized certification tests of tractor ROPS are today performed by various organizations according to internationally standardized static procedures, such as: the Organisation for the Economic Cooperation and Development (OECD), the International Organization for Standardization (ISO) and the European Community (EC). Unfortunately, given their age and language of publication, not all the works of Moberg [5], Chisholm [6,7,8,9] and Schwanghart [10,11] are readily accessible. This has hindered the ability of contemporary researchers to revisit the underlying assumptions and the original tractor parameters used in those early studies. It is thus difficult to determine the applicability of those works to modern tractors. 

Current standard procedures define the required energy to be absorbed by tractor ROPS frames/cabs during a sequence of loadings (see, for example, [12]). Recent debate has arisen in relation to the rollover dynamics and, specifically, whether or not the load sequence reflects the most common roll progression and energy magnitudes and if the effect of strain rate in a real rollover event is accounted for by the static procedure; these have important implications for the driver’s survival volume [1,13] and dynamic loading. Additionally, the tractor’s reference mass (*M_r_*), defined by the tractor manufacturer and often close to the unladen mass, governs the test’s load calculation. The driver safety margin provided by these standardized procedures is dependent on the ratio between the reference mass and the maximum laden mass of modern tractors [14,15]. This uncertainty is further confounded if the evolution brought about by the changing role of the tractor (e.g., variable permissible track widths, addition of front-end-loaders, etc.) in modern agriculture with respect to the laden operating mass is considered. 

Despite acknowledgement that tractor designs, and hence their physical parameters and role in farming, have changed since the standardized processes were developed [16,17], there are few investigations of rollover behavior using modern tractors [18,19]. There is, thus, some uncertainty in knowing how the rollover energy is partitioned to the ROPS and other components during contemporary, real rollover events corresponding to common modern tractor configurations. In addition, a recent issue that has arisen at the international level refers to the adoption of foldable ROPSs and their incorrect use among users responsible for the relevant percentage of the fatal accident occurring with tractors [20,21,22,23,24,25]. Hence, although the pioneering works are fundamental to the establishment of the standards, the applicability of the resulting standardized procedure to today’s diverse tractor fleet and configurations is somewhat unknown. This is reflected by the Standardisation Committee [26] recognition that the only means to investigate the rollover energy present in modern tractor rollover is to conduct actual rollover tests with modern tractors [1,18,27,28]. 

Nowadays, as modern tractors are often equipped with silent-block and many different tire options to match with farming operations where crops and environments can highly differ, the aim of this paper is to investigate the energy dissipated during controlled lateral rollover of a modern narrow-track tractor mounted with the two extremes of permissible tire configurations (wide and narrow) and ROPS silent-block suspended cab. Leveraging the original research approaches of Chisholm [6,7,8,9] and Schwanghart [10,11], testing was used to evaluate tire energy dissipation in tractor rollover to better understand the actual energy absorbed at the ROPS cab level. A wide tire configuration and a narrow tire configuration were investigated and the effect of the silent-blocks isolated from the ROPS system. Here, wide/narrow refers not only to the tire widths but so too the track width. The wide configuration was selected because, consistent with the T1 tractor category of the EU tractor type definition [15], it is typical of open field operations, while the narrow one, equivalent to the T2 tractor type represents orchard/vineyard configuration.

## 2. Materials and Methods

### 2.1. Experiment Design

**Figure 1:**
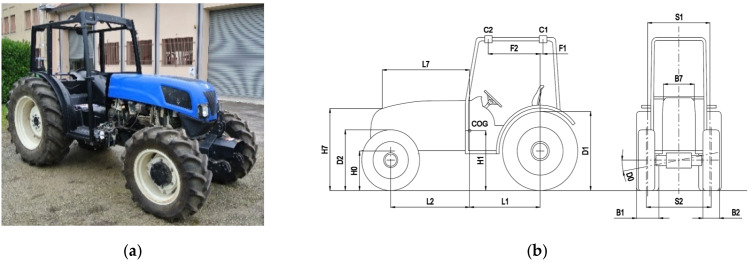
Tested tractor (with non-structural cab components removed) (**a**) and main physical parameters according to Table 1 (**b**).

The tests in this study were conducted at the official OECD Test Station, Laboratorio di Meccanica Agraria, of the Alma Mater Studiorum, University of Bologna. A New Holland narrow-track tractor, TN 4000F, specifically designed by CNH for orchard work, was used (Figure 1). The tractor model was fitted with a ROPS cab frame tested in accordance with OECD Code 7 [29]. Given its specified reference mass of 3000 kg, according to the official ROPS testing procedure (*E* = 1.75 *M_r_*), the lateral load energy value to be reached is 5250 J. In preparing the tractor for the rollover tests, the plastic roof, glass and exhaust extension were removed as they were considered non-structural components of the ROPS cab and were likely to be unnecessarily damaged during the tests. Furthermore, it was assumed that these components could assist in the dissipation of rollover energy and hence, their removal would likely lead to more energy being subjected to the ROPS, thus providing a less conservative test. It was recognized that the contribution of tires to the energy dissipation could vary depending on their features, tractor geometry and the dynamics of the particular rollover event. To provide insight into the tire contributions, controlled tractor lateral rollovers were planned with two different tire configurations: 

(T1) rear tire type 420/70R30 (134A8), with a cross-section of 418 mm and overall diameter of 1398 mm and front tire type 320/70R20 (113A8), with a cross-section of 319 mm and overall diameter of 0.982 mm. This resulted in a rear external base width of 1620 mm and a front external base width of 1568 mm;

(T2) rear tire type 270/95R36 (139A8), with a cross-section of 275 mm overall diameter of 1428 mm, and front tire type 11.2R24 (114A8), with a cross-section of 284 mm overall diameter of 1095 mm. This resulted in a rear external base width of 1290 mm and a front external base width of 1314 mm.

Table 1 reports the relevant physical parameters for the T1 and T2 configurations of the tested tractor. The Center Of Gravity (COG) height and inertia for each configuration were determined using an oscillating platform [30]. Parameters reported in Table 1 clearly evidence the effect of tire selection on the main features of the tractor which influence tractor stability and dynamic performance.

**Figure 2:**
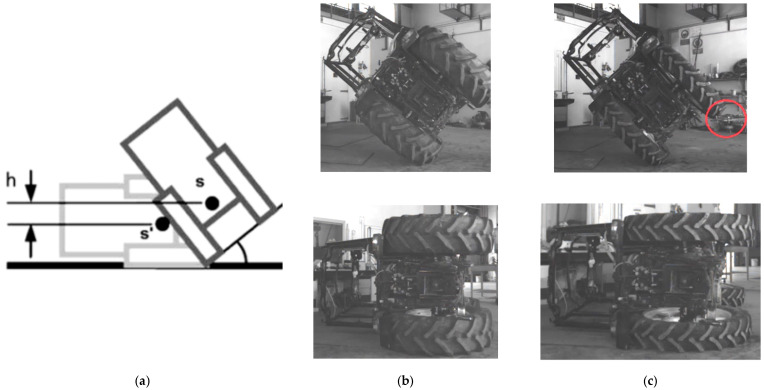
Lateral tractor rollover onto a horizontal surface: (**a**) Schematic representation where **s**, **s’** indicate the COG initial and rest positions respectively; actual tested tractor in T1 (**b**) and T2 (**c**) configuration.

Fundamentally, a tractor rolls from an unstable state (e.g., when the COG exits the base) to a stable state (e.g., the ground surface), as depicted in Figure 2a. The T1 and T2 configurations evaluated in the rollover tests are shown in Figure 2b,c. Ignoring any initial velocity, tire deformation or dissipation mechanisms, it can be assumed that the available energy is related to the potential energy between the initial and the rest positions [27]:

*E_C1_* = *mgh* (1)

where *m* corresponds to the tractor mass, *g* is the gravity acceleration and *h* is the COG vertical position variation. 

The technique conceived to generate lateral tractor rollover with minimal external input loading involved elevating the contact patch of the rear wheel with a conventional forklift. A tethered sliding steel plate was mounted between the forks and the tire to minimize the lateral tire-fork force (i.e., friction force) at the tire contact zone by generating a sliding metal-metal interface (see Figure 2c, red circle). The forks and, hence, tire were elevated quasi-statically until the unstable equilibrium position was reached. At this point, the tractor laterally rolled onto the horizontal floor. Prior to the test, the impact zone of the concrete floor was covered with a 12 mm steel plate in order to define a rigid surface and remove the likelihood of cracking the concrete floor. This arrangement likely subjected the cab to a more severe impact (i.e., by decreasing the impact time). Three tests were planned for each configuration to verify repeatability. 

### 2.2. Data Acquisition

**Figure 3:**
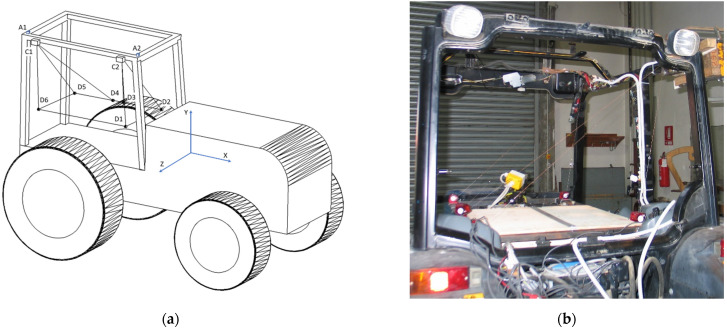
Schematic representation of the instrumentation fitted on the tractor showing the accelerometers, A1 and A2, the load cells, C1 and C2, and the linear displacement transducers, D1–D6, connected to the load cells (**a**) and actual system (**b**).

Table 2 lists the instrumentation fitted on the tractor to enable measurement of the rollover data in the controlled tests. The location and orientations of the equipment are shown in Figure 3. The ROPS force and displacement were measured at the points of contact with the ground in order to permit the energy absorbed by the ROPS frame to be quantified. The strain energy absorbed can be obtained assuming that:

(2) $$E=\overset{x_{2}}{\underset{x_{1}}{\int }}Fdx≅\;\overset{n}{\underset{i=1}{\sum }}F_{i}\left(x_{i}-x_{i-1}\right)$$

where *F* is the force measured by the dynamic load cell and *x* is the deflection measured by the linear dynamic transducer.

Energy due to wave propagation within the structure and floor (steel plates) [31], resulting from the loads cells impacting the rigid surface, was not accounted for. 

Two dynamic triaxial piezoelectric load cells were mounted rigidly to the vertices of the upper right-hand side of the cab frame (Figure 3). The free surface of each cell had a metal block rigidly attached to protect the cell and to provide the contact surface with the floor. The outer surface of each block was ground with large radii to ensure that when the blocks/cells contacted the ground, minimal moment was subjected to them. Six linear displacement transducers, three connected to a point on the *z* axis of each load cell, were arranged to measure the trajectory of each transducer in 3D space relative to a frame rigidly connected to the cab floor (Figure 3). Load cell rotation was not accounted for as the error due to this effect was assumed to be minimal as a result of the profile of the ground block and the expected displacement due to the mounting on the cab frame. The widths of the symmetric cab where the two force transducers, C1 and C2, were placed were 1055 mm and 1000 mm, respectively. Each load cell protruded an additional 98 mm. The distance between the center of the two cells was 892 mm. Prior to commencement of the lateral rollover tests, static tests were carried out in order to render the instrumentation set-up feasible [28].

A high-speed video camera (Table 2) was used to record the rollover tests. This camera was positioned on a tripod located at a distance to provide a means for cross-checking rollover behavior. The tripod feet were placed on sponge foam to isolate the camera from shock vibration transmitted through the floor (steel plates) as a result of the impact. Given the high frame rate (1000 fps) and hence very low exposure, additional lighting was provided using spot lights focused on the tractor and impact zone. The individual frames from the video record were used to obtain the potential and kinetic energy information. In order to do this, reflective tape markers were affixed to rigid parts of the tractor chassis. The distance between them was accurately measured. The camera software, AOS Image Studio (AOS Technologies AG, Baden Daettwil, Switzerland), permitted the change in position of the reflectors to be measured frame-by-frame to calculate the tractor COG trajectory during the tractor rollover. Using the known video camera frame rate, the linear and angular velocities for the tractor rigid body were determined using the instantaneous velocity of at least two reflector locations in each frame. The data acquisition was performed using custom developed LabView 8.2 software (National Instruments Corporation, Austin, TX, USA). The software was developed with the facility to start acquisition from all devices at the same time to ensure synchronous measurement and aid post processing of the data. 

### 2.3. Trilateration of Load Cell Trajectory

The procedure developed for recovering the deformation of the ROPS cab involved measuring the path trajectory of the load cells during the tests with respect to the cab base and was based on trilateration.

**Figure 4:**
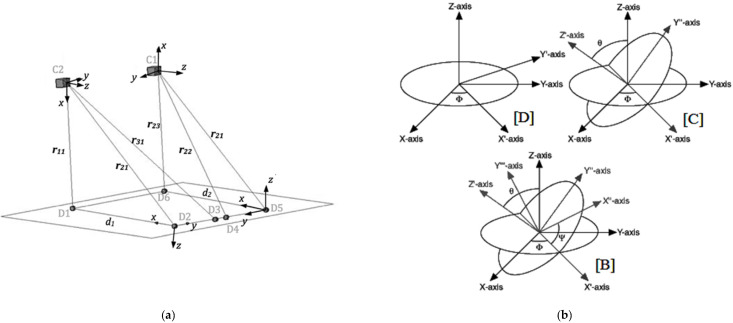
Details of trilateration method used to measure z displacement of each cell relative to cab base: (**a**) Triaxial force transducers (load cells, C1 and C2) with linear displacement transducers (D1–D6) connected; (**b**) Coordinate rotation with the Euler angles, where [D] rotation about Z-axis by φ, [C] rotation about X′-axis by θ, [B] rotation about Z′-axis by ψ.

With reference to Figure 3a, each load cell position is represented by the intersection of three vectors with their respective origins located at D1, D2 and D3 for the load cell C2 and D4, D5 and D6 for the load cell C1. Mathematically, given only the length information, each vector defines a sphere with its center at the vector origin. Linear algebra [32] dictates that the intersection of three spheres with centers in the same plane occurs at two points. To simplify the analysis, it was assumed that one sphere had its center at *x* = 0 and another had its center on the *x*-axis, thus:

(3) $$x_{ni}=\frac{r_{n_{1i}}^{2}-r_{n_{2i}}^{2}+d_{n}^{2}}{2d_{n}}$$

(4) $$y_{n_{1}}=\frac{r_{n_{1i}}^{2}-r_{n_{3i}}^{2}+\alpha_{n}^{2}+\beta_{n}^{2}}{2\beta_{n}}-\frac{\alpha_{n}}{\beta_{n}}x_{ni}$$

(5) $$z_{ni}=\pm \sqrt{r_{n_{1i}}^{2}-x_{ni}^{2}-y_{ni}^{2}}$$

where *x_ni_*, *y_ni_*, *z_ni_* correspond to the local coordinates of each 3-vector (*r_n_*) system and *α*_n_ and *β*_n_ are the *x* and *y* coordinate values of transducers D3 and D4, respectively. The other quantities can be inferred from Figure 4a. Depending on the orientation of the coordinate frame, it is decided whether to accept the positive or negative value generated by Equation (5). A model was developed in LabView 8.2 (National Instruments) characterized by 3D Cartesian Coordinate Rotation, based on Euler rotation theory (Figure 4b). Equation (6) allows the local position and force information to be orientated in a consistent framework which is necessary for subsequent force-displacement calculation purposes and hence, the work, and thus, energy calculation:

(6) $$\left[\begin{array}{c} X_{ni}\\ Y_{ni}\\ Z_{ni} \end{array}\right]=A^{"}\left[\begin{array}{c} x_{ni}\\ y_{ni}\\ z_{ni} \end{array}\right]$$

The left hand column vector of Equation (6) provides the position information for each cell in tractor cab base coordinates for a reference system fixed to the cab floor, as shown in Figure 3. According to Equation (6), these data are obtained via multiplication of the local position information with a matrix *A*″. Matrix *A*″ is comprised of Euler angle rotations (Figure 4b) specific to the particular case and coordinates in question. The same procedure was followed to rotate the load cell force axis coordinates to be parallel to the cab base coordinates. As an example, to make the local C2 coordinates perpendicular to the base system, the standard Euler angle multiplication was performed to generate the effective *A*″ matrix for that case, with *A*″ = *BCD*, Equation (7).

(7) $$D=\left[\begin{array}{ccc} \cos\varphi & \sin\varphi & 0\\ -\sin\varphi & \cos\varphi & 0\\ 0 & 0 & 1 \end{array}\right],\;C=\left[\begin{array}{ccc} 1 & 0 & 0\\ 0 & \cos\theta & \sin\theta \\ 0 & -\sin\theta & \cos\theta \end{array}\right],\;B=\left[\begin{array}{ccc} \cos\psi & \sin\psi & 0\\ -\sin\psi & \cos\psi & 0\\ 0 & 0 & 1 \end{array}\right]$$

### 2.4. Silent-Block Support System

**Figure 5:**
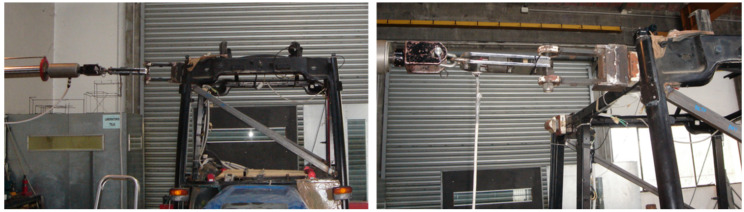
Dynamic test set-up showing tensile loading of the C2 load cell using the magnetic pliers.

Silent-blocks are used on tractors primarily to improve driver comfort through better Noise Vibration and Harshness (NVH) performance. The ROPS cab frame was mounted on the tractor in four locations via a silent-block arrangement. The two front blocks were the same model and the two rear blocks were the same model, however, the front and rear block models were different. Silent-block stiffness properties related to the vertical movement of the cab are typically provided by the supplier so that the tractor manufacturer can tune the cab NVH characteristics. However, in regard to rollover, their effective lateral stiffness and damping properties affecting their energy storage and dissipation, respectively, are not readily available. Furthermore, it should be noted that the static tests foreseen by the international ROPS testing codes require that the tractor chassis be rigidly secured. The force-displacement of the ROPS system, comprised of the cab and its attachments to the chassis via the silent-blocks, thus, is measured as one entity. The force-displacement measurement and data acquisition system developed in this study permitted the behavior of the ROPS cab contact points relative to the cab base to be known. In order to evaluate the ROPS system with respect to the tractor, it was necessary to characterize the effect of the silent-blocks in lateral rollover. To this end, both static and dynamic tests on the cab were performed. All tests were repeated three times to gauge repeatability. The static tests were performed using the standard ROPS loading system available at the official OECD Test Station, Laboratorio di Meccanica Agraria, of the Alma Mater Studiorum, University of Bologna. The system is based on a hydraulic cylinder fitted with a load cell and a linear displacement transducer to measure the force and ROPS deflection under loading. The tractor was rigidly secured to the ground and the tests performed with separate lateral forces (i.e., axially to the cell) applied at the load cells’ C1 and C2 locations. Forces were applied in this direction since this was representative of the primary force direction during lateral rollover. It was necessary to reinforce the cab so as to ensure that the measured displacement was due to the silent-blocks alone and not the cab frame deformation. The static tests were performed post rollover and hence, the cab reinforcements were not present in the rollover tests. Using the video camera (Table 2), the local deformation of the silent-blocks was observed in order to determine when contact with their end-stop had been reached. The force-displacement information was recorded. To evaluate the lateral damping properties of the silent-blocks, the vibration characteristics of the reinforced cab had to be measured. This was achieved using the accelerometer attached to the cab floor (Table 2) and measuring the free vibration signal. Due to the inherent difficulties associated with instantaneously removing the loading cylinder when applying a compressive load, it was decided to apply a tensile load via a quick release mechanism achieved through using a set of magnetic pliers, with a maximum tensile force capacity of 5000 N (Figure 5). Since vibration would occur about the equilibrium position, it was assumed that the lateral damping was symmetrical (i.e., the same in compression as in tension). As a first approximation, a single degree-of-freedom (1DOF) mass-spring-viscous damper model was fitted to the filtered measured data. The effective damping ratio for the front and rear vibrations were determined via the log decrement method [33]. 

## 3. Results

**Figure 6:**
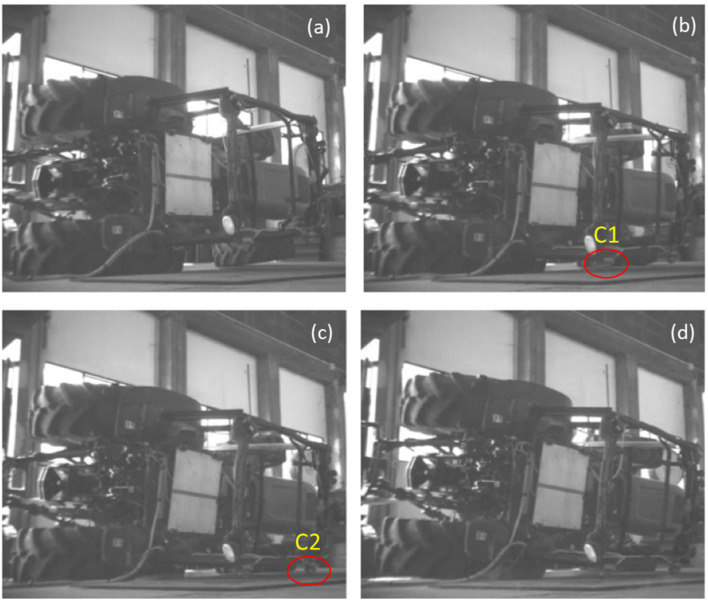
Typical roll sequence for the T1 configuration: (**a**) Complete rear tire contact (no ROPS contact); (**b**) Just prior to C1 contact (large tire deformation); (**c**) Just prior to C2 contact (large tire deformation); (**d**) Release of tire elastic energy.

The sequence of tests was carried out on the same ROPS with the T1 configurations tests conducted first. Although three tests for each configuration were scheduled, only two tests in the T2 configuration were possible. This was unfortunately due to the destructive nature of the testing; the greater amount of available energy and lesser means for tire dissipation in T2 subjected more energy to the ROPS in this configuration resulting in the premature termination of the testing. Using a new cab frame for each test was not feasible due to economic constraints associated with both cab hardware and labor required for cab removal and mounting. An example of how the tires behaved during a lateral rollover onto the rigid horizontal surface is shown in Figure 6. The configuration corresponds to that of T1 and the initial position is that shown in Figure 2b. The presented frames show the different contributions of the rear tire. Qualitatively, it can be noted that: (Figure 6a) with complete tire wall contact and the tractor in a near horizontal position the ROPS is yet to make contact with the surface; (Figure 6b) just prior to C1 contact there is large deformation of the rear tire; (Figure 6c) just prior to C2 contact the large tire deformation is still observable, and (Figure 6d) the release of some of the tire elastic stored energy is revealed by the slight lifting of the rear end and of rear tire compression. The amount of dissipation depends on the tire damping properties [9,34]. 

**Figure 7:**
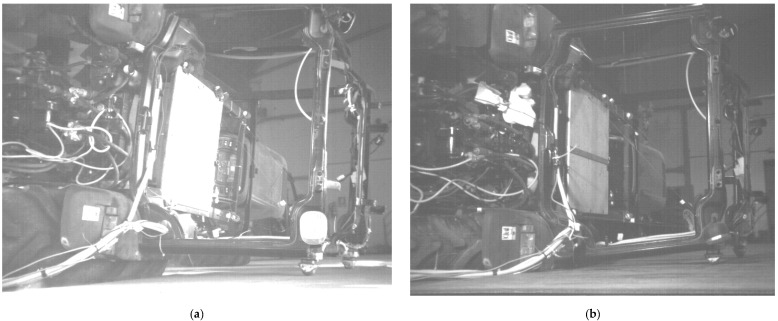
Close-up views of ROPS contact for the: **T1** wide configuration (**a**), and **T2** narrow configuration (**b**).

The T2 configuration (Figure 7) caused the ROPS to contact the ground sooner than the T1 configuration. This results in less percentage of energy being dissipated by the tires. It should be again noted that the tire wall protruded more from the rim in the T1 configuration. It is likely that this increases the tire’s ability to absorb energy. Examination from the video records suggested that the narrow configuration T2 absorbed and dissipated less energy in the tire. As this modern tractor cab was fitted to the chassis via silent-blocks and the energies measured by the trilateration were the result of the force-displacement information measured at the contact points of C1 and C2 on the ROPS frame with respect to the cab base, the dynamic energy dissipation from the blocks was not directly captured. 

### 3.1. ROPS Energy Absorption

**Figure 8:**
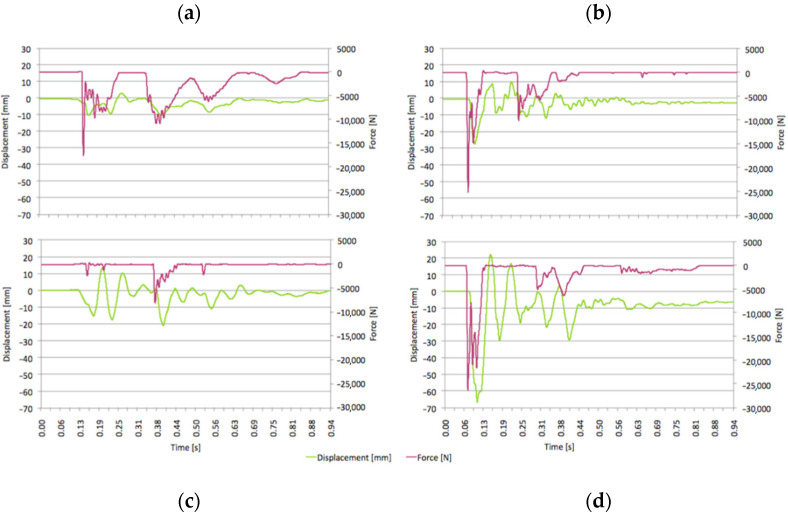
Representative globally transformed Z -axis direction force-displacement results corresponding to **C1** for each test in the **T1** (**a**) and the **T2** (**b**) configurations and to **C2** for each test in the **T1** (**c**) and the **T2** (**d**) configurations.

Figure 8 displays representative examples of the force-displacement measurements corresponding to each load cell for the T1 and T2 tests. It is apparent that the load cells, and hence, upper corners of the cab, impact the ground multiple times and that the cab frame vibrates as a result. The results are for the measured data transformed into the global *Z* axis orientated effectively in the near normal contact direction using the trilateration method described previously. Similar procedures were adopted to manipulate the data for the *X* and *Y* directions. These values are not graphically presented here as they represent less crucial information. Their respective energy values, however, were included in the calculation of the total energy. The average total energy *E_TOT_*, dissipated through work, calculated according to Equation (2) for the T1 and T2 tests, were found to be 460 J and 1990 J, respectively. It is thus inferred that as far as the ROPS cab energy absorption requirements are concerned, the T1 test is less demanding than the T2 type test. This is demonstrated by the smaller forces and displacements (and hence energy). In the T2 (narrow) configuration, the ROPS width was much closer to the rear external tire width. Additionally it should be noted that the tire wall protruded less from the rim in this configuration. As such, there was less opportunity for the tire to absorb energy during the lateral rollover prior to making rim contact. Rim contact was confirmed via visual inspection of the rims/front hub after each test which clearly indicated damage. It was also observed that the change in configuration led to more synchronous impacting of the ROPS at C1 and C2 for the T2 configuration. The force-displacement behavior of T2 shows greater impact energy. Plastic deformation of the ROPS is also demonstrated by the difference in displacement prior to and once the ROPS cab has essentially stopped vibrating.

### 3.2. Silent-Blocks’ Energy Absorption

**Figure 9:**
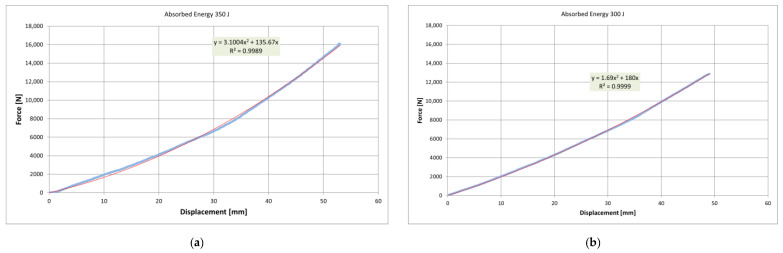
Effective silent-block force-displacement characteristics for static tests performed on: (**a**) Load cell (C1); (**b**) Load cell (C2). Blue is the measured result and red is the best fit quadratic forced through the origin.

**Figure 10:**
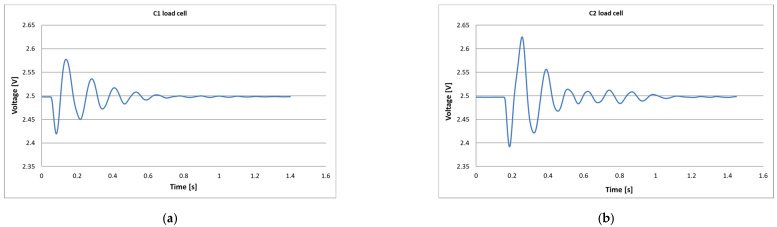
Effective silent-block behavior for dynamic tests performed on: (**a**) Load cell (C1); (**b**) Load cell (C2).

The static characterization of the silent-blocks upon reaching their end-stop limit of travel, showed that when the force was applied to the C2 front cell and then to the C1 rear cell 350 J (Figure 9a) and 300 J (Figure 9b), respectively, were absorbed. Scope exists to infer energy dissipated during rollover events through measuring ζ values before and in the cab motion during rollover. Since ζ < 1, the system is considered underdamped laterally [35]. The dynamic characterization using the 1DOF model implied damping ratios (ζ) of 0.13 for the front and 0.11 for the rear silent-blocks treated collectively (Figure 10).

### 3.3. Tractor Available Energy

**Figure 11:**
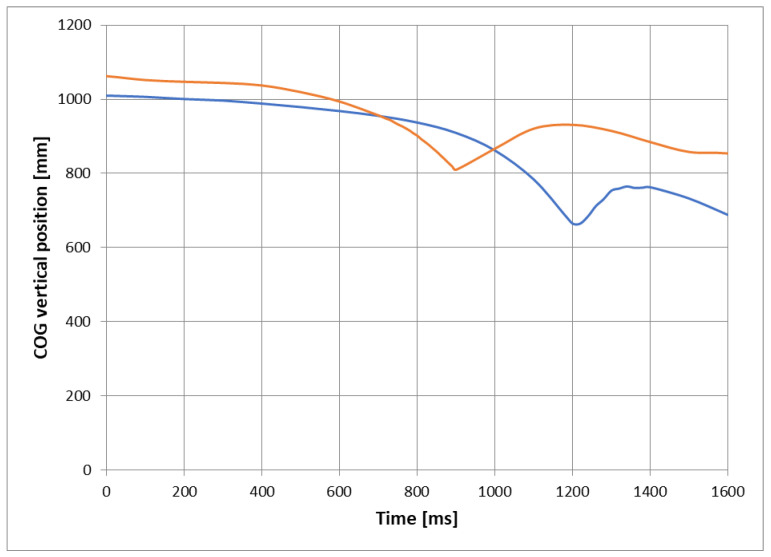
Actual COG vertical position for T1 (**blue line**) and T2 (**red line**) configurations during the rollover test.

The data from Table 1 was used to calculate the theoretically calculated energy *E_C1_*, as shown in Equation (1), for the T1 and T2 configurations giving 9623 J and 11,780 J, respectively. The greater amount of available potential energy in the narrow configuration (T2) is a result of both raising the COG and reducing the track width (Table 1): both effects increase h (Figure 2a) from 0.328 m to 0.402 m. The interpretation of *E_C1_* is straightforward and requires no additional explanation of the method. *E_C2_* is remarkable. Using the video technique, it was possible to determine more accurately the actual COG position (Figure 11) and the available energy and account for tire deformation and initial kinetic energy due to non-zero COG velocity. Based on the frame-by-frame analysis explained previously, the values recovered from this method indicated the actual available energy *E_C2_* to be 7479 J for case T1 and 10,410 J for case T2. These values were 78% and 88%, respectively, of those calculated theoretically (9623 J and 11,780 J). 

**Figure 12:**
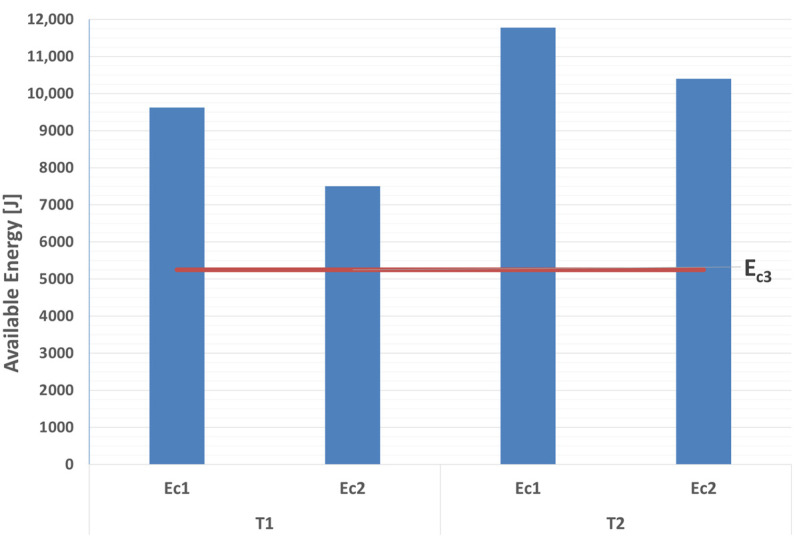
Energy values E_C1_, E_C2_, E_C3_ for T1 and T2 configurations.

The energy values, calculated according to the height of the COG, Figure 2a, denoted *E_C1_*, and that inferred from the video records accounting for both the initial potential and kinetic energies, *E_C2_*, are depicted in Figure 12 along with the OECD standardized energy *E_C3_* of 5250 J [29]. 

One would expect the values to be different since, in the rollover initiation position, the total mass of the tractor is supported by the side front and rear tires, which are not infinitely stiff; the video record revealed that the wider tire reduced the height values more so than the narrower ones due to the tires being less stiff. In the actual rollovers, the front pivot point was also left mobile. Both effects reduce the COG height value of the initiation position and hence reduce the available energy. The non-zero initial velocity components in each case were very small and, despite being included in this work-energy analysis, contributed insignificantly to the total.

## 4. Discussion

**Figure 13:**
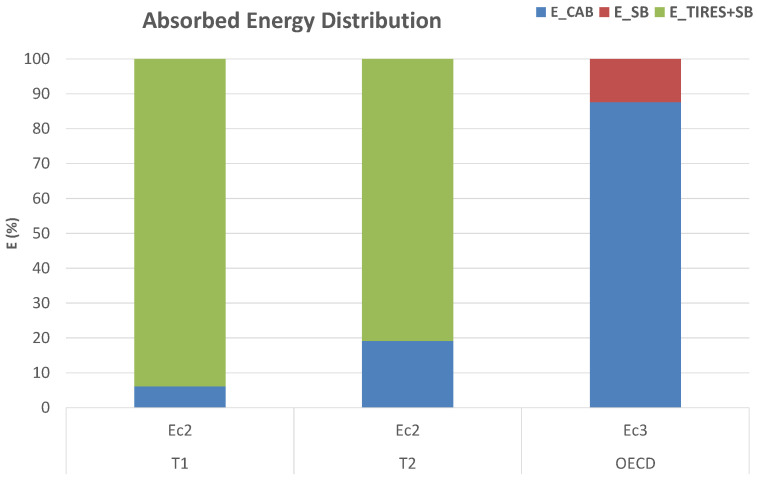
Energy *E_C2_* values for T1 and T2 configurations compared with the OECD test energy *E_C3_*.

The comparison to the OECD test energy *E_C3_*, as depicted in Figure 12, is worth discussing. Although the specified ROPS energy equates to 100%, part of that system comprises the energy absorbed by the silent-blocks. As can be seen from the results, the energy absorbed at the ROPS contact points with respect to that actually available *E_C2_* for the tests in configuration T2 was higher than that of configuration T1. Ignoring the potential for other tractor parts to contribute to the energy dissipation [27], Figure 13 shows the energy E_C2_ for the T1 and T2 configurations, with E_CAB being the energy absorbed by the ROPS cab, E_TIRES+SB being the remaining energy assumed to have been absorbed by the silent-blocks and tires during the multiple ROPS-ground impacts. The experimental tests hence demonstrate the significant contribution of the tires in the energy dissipation as alluded to by Chisholm [6,7,8,9] and Schwanghart [11] and warrant the need for more work on tire and silent-block dynamic energy absorption. It is noted that the OECD standardized test does not foresee a tire contribution in the energy dissipation during the cab static test.

E_CAB is the energy absorbed by the cab, E_SB is the sum of the static energy absorbed by the silent-blocks, E_TIRES+SB is the remaining available energy assumed to be primarily dissipated by the tires and silent-blocks during the multiple cab-ground impacts.

## 5. Conclusions

This work investigated the energy absorbed by a modern narrow-track tractor ROPS during lateral rollover when configured at its permissible wide (T1) and narrow (T2) track arrangements. The tractor selected for the case study was the New Holland TN 4000F fitted with a cab type ROPS mounted to the chassis via silent-blocks. The percentage of energy absorbed by the ROPS cab frame at the contact points was relatively small with respect to the available energy in the tractor lateral rollover. This was especially observed for the wide configuration (T1) in which much energy was absorbed and likely dissipated by the tire assembly. Calculating the available energy based on tractor geometry with rigid tires and no front axle pivot overestimated the available energy. Indeed, the evaluation of the video records indicated that the effect of tire deformation and front axle pivot should be included in energy measurements and rollover models. Silent-blocks fitted to the ROPS were also considered. Their lateral static and dynamic characterization could represent a valuable element in the ROPS design and development due to their role in the official ROPS strength tests and actual rollover dynamics.

The measured results indicate that in both tested cases, there was a safety margin in the lateral rollover with respect to the applied energy during the official OECD procedure of lateral loading of the ROPS. Whether or not this margin provides an acceptable safety level to the tractor operator depends on the reliability of a direct correlation between static and dynamic absorbed energies and impact forces, particularly those subjected to the operator, and this requires further investigation.

Nevertheless, the current coded ROPS procedures, being standardized approaches necessitating a simplified testing process which is straightforward to implement and generates repeatable results, refer to energies to be absorbed by the ROPS, calculated on the basis of the tractor mass. Given the highlighted results in this paper, it seems advisable to include in the coded procedures the tractor’s physical parameters belonging to the worst case of tire configuration for the specific tractor-ROPS combination.

As shown, this rollover experiment performed on a modern tractor equipped with two tire configurations clearly demonstrated that tire selection affected not only the amount of energy absorbed at the tire level during the rollover event but also the potential energy available when the tractor reaches the unstable equilibrium.

Tractors are designed to be multipurpose machines which appeal to a wide range of potential user groups and, consequently, different sets of tires are available for the normal operations in field. Additional activity is necessary to help manufacturers and testing authorities identify and accommodate the tire set representing the most critical condition during a rollover event to ensure that manufacturers’ design and authorities certify ROPS that perform their intended function of preserving the operator’s life irrespective of tire configuration.

## Figures and Tables

**Table 1:** Main physical parameters of the modified tested tractor in the T1 and T2 configurations.

Tractor Parameter	T1 Value	T2 Value	Unit
Rear tire size	420/70R30	270/95R36	
Front tire size	320/70R20	11.2R24	
Rear tire overall diameter (D1)	1.398	1.428	M
Front tire overall diameter (D2)	0.982	1.095	M
Rear external base (B1)	1.620	1.290	M
Front external base (B2)	1.568	1.314	M
Rear wheel track (S1)	1.202	1.015	M
Front wheel track (S2)	1.249	1.030	M
Wheelbase (L1 + L2)	2.450	2.450	M
Engine bonnet width (B7)	0.450	0.450	M
Engine bonnet height (H7)	1.310	1.366	M
Front-axle swing angle from zero position to end of travel (D0)	0.140	0.140	Rad
Height of front-axle pivot point (H0)	0.600	0.656	M
Height of COG (H1)	0.795	0.828	M
Horizontal distance COG—front-axle (L2)	1.436	1.427	M
Horizontal distance COG—rear-axle (L1)	1.014	1.023	M
Horizontal distance between the COG and the front corner of the engine bonnet (L7)	1.520	1.520	M
Moment of inertia about the longitudinal axis through the COG (I)	673.6	608.7	kg m^2^
ROPS height at the point of impact (C1 cell)	2.119	2.123	M
ROPS height at the point of impact (C2 cell)	2.150	2.175	M
Horizontal distance C1—rear-axle (F2)	0.038	0.038	M
Horizontal distance C2—rear-axle (F1)	0.930	0.930	M
Tractor mass (m)	2990	2988	Kg

**Table 2:** Data acquisition system components of the test tractor.

REF	QTY	Instrument	Purpose	Specifications
A1, A2	2	3DOF piezoelectric accelerometers	measure components of acceleration in two locations	acc. range ± 500 g
C1, C2	2	triaxial dynamic load cell	measure 3 perpendicular components of force	range Fx, Fy ± 20 kN, Fz ± 40 kN
D1-D6	6	cable type linear dynamic 3 3 connected to each force transducer	measure displacement	range 228.6 mm max. cable acc. 136 g
-	1	onboard data acquisition unit	record accelerometer reading in the time domain	65.536 kHz max. sampling rate
